# Platelet-derived growth factor-A regulates lung fibroblast S-phase entry through p27^kip1^ and FoxO3a

**DOI:** 10.1186/1465-9921-14-68

**Published:** 2013-07-02

**Authors:** Stephen E McGowan, Diann M McCoy

**Affiliations:** 1 Department of Veterans Affairs Research Service, University of Iowa Carver College of Medicine, Iowa City, USA; 2 Department of Internal Medicine, University of Iowa Carver College of Medicine, Iowa City, Iowa, USA; 3 Division of Pulmonary, Critical Care, and Occupational Medicine, C33B GH, Department of Internal Medicine, University of Iowa Hospitals and Clinics, 200 Hawkins Dr., Iowa City, Iowa, 52242m, USA

**Keywords:** Alveolar development, Emphysema, Pulmonary fibrosis, Cell cycle-regulation, Secondary septation, Platelet-derived Growth factor Receptor-alpha

## Abstract

**Background:**

Secondary pulmonary alveolar septal formation requires platelet derived growth factor (PDGF-A) and platelet derived growth factor receptor-alpha (PDGFRα), and their regulation influences alveolar septal areal density and thickness. Insufficient PDGFRα expression in lung fibroblasts (LF) results in failed septation.

**Methods:**

Mice in which the endogenous PDGFRα-gene regulates expression of the green fluorescent protein were used to temporally and spatially track PDGFRα-signaling. Transition from the G_1_/Go to the S-phase of the cell cycle was compared in PDGFRα-expressing and non-expressing LF using flow cytometry. Laser scanning confocal microscopy was used to quantify p27^kip1^ and forkhead box "other" 3a (FoxO3a) in the nuclei of alveolar cells from mice bearing the PDGFRα-GFP knock-in, and p27^kip1^ in mice with a conditional deletion of PDGFRα-gene function. The effects of PDGF-A on the phosphorylation and the intracellular location of FoxO3a were examined using Western immuoblotting and immunocytochemistry.

**Results:**

In neonatal mouse lungs, entry of the PDGFRα-expressing LF subpopulation into the S-phase of the cell cycle diminished sooner than in their non-expressing LF counterparts. This preferential diminution was influenced by PDGFRα-mediated signaling, which phosphorylates and promotes cytoplasmic localization of FoxO3a. Comparative observations of LF at different ages during secondary septation and in mice that lack PDGFRα in alveolar LF demonstrated that nuclear localization of the G_1_ cyclin-dependent kinase inhibitor p27^kip1^ correlated with reduced LF entry into S-phase.

**Conclusions:**

Nuclear localization of FoxO3a, an important regulator of p27^kip1^ gene-expression, correlates with diminished proliferation of the PDGFRα-expressing LF subpopulation. These mechanisms for diminishing the effects of PDGFRα-mediated signaling likely regulate secondary septal formation and their derangement may contribute to imbalanced fibroblast cell kinetics in parenchymal lung diseases.

## Background

During the period of maximal secondary alveolar septal elongation, which extends from postnatal day 4 (P4) through day 14 in mice, the proliferation of interstitial lung fibroblasts (LF) declines from its initial maximum. Our prior studies have shown that platelet-derived growth factor receptor- (PDGFR) kinase activity sustains proliferation and prevents apoptosis of PDGFRα-expressing LF [1]. We also observed that septal thinning, which primarily occurs after P12, was accompanied by interstitial cell apoptosis that was tempered by PDGFR-kinase activity [1]. These findings suggest that a reduction in PDGFRα-mediated signaling may regulate thinning at the end of septal elongation. We have now investigated potential mechanisms whereby diminished PDGFRα-signaling decreases cell cycle progression in LF, which express this receptor and are critical for secondary septal formation.

PDGF-A, and its exclusive signaling through PDGFRα, are absolutely required for secondary alveolar septal formation [2]. PDGFRα-expressing LF preferentially localize to the distal portion of the septa, contain more abundant ACTA2 (α-smooth muscle actin, αSMA), and are required for elastic fiber formation [3]. In neonatal rats, the ^3^H-thymidine autoradiographic labeling index of interstitial LF was maximal at P4 and declined afterwards, and cells nearest the origin of the secondary septal crests were more likely to proliferate [4]. Although the labeling index declined, the number of ^3^H-labeled LF remained elevated until P10, after which it also declined. Sub-populations of neonatal LF have been distinguished based on the abundance of cytoplasmic lipid droplets or the presence of the surface glycoprotein thymocyte differentiation antigen-1(Thy-1) [5]. The labeling index of lipid-laden LF declines earlier than for LF which lack lipid droplets [6]. Similarly, the proliferation of LF which contain αSMA, and more abundantly express PDGFRα, declines earlier than for LF which do not express PDGFRα [7].

Because LF progression through the G_1_/S phase of the cell cycle is linked to PDGFRα-kinase activity, we hypothesized that the progression is determined by downstream phosphorylation targets of PDGF-A, more specifically those involving Akt. Our prior studies demonstrated that PDGF-A stimulates the phosphorylation of Akt in primary cultures of neonatal mouse LF. PDGFRα primarily signals through the PI3K pathway, for which a major downstream target is Akt/protein kinase B [1]. After cytoplasmic phosphorylation by PI3K, Akt can translocate to the nucleus and phosphorylate the cyclin-dependent kinase (CDK) inhibitor 1B (p27^kip1^), which is then more likely to bind the chaperone protein 14-3-3 and be exported to the cytoplasm, where it is degraded. pAkt is cleared from the nucleus in the presence of promyelocytic leukemia (PML) bodies and this process is augmented by the phosphatases phosphatase and tensin homologue (PTEN) and protein phosphatase 2A (PP2A) [8].

Nuclear translocation also regulates the function of Forkhead box factor O (other) transcription factors (FoxO) [9]. Akt can phosphorylate FoxO3a and thereby regulate its ability to promote *p27*^*kip1*^ gene transcription [9,10]. FoxO3a primarily localizes to the nucleus where its effects on transcription are increased by acetylation and association with members of the HDAC complex [9]. Akt can phosphorylate three residues on FoxO3a: threonine (T)24, serine(S) 253 and S319; and S253, which is located in the nuclear localization domain and has the greatest impact on DNA-binding and FoxO3a translocation [9]. Progressive phosphorylation reduces the stability of FoxO3a-DNA interactions, promotes complex formation with 14-3-3, and thereby increases cytoplasmic export [11]. Phosphorylated cytoplasmic FoxO3a is ubiquitinated and degraded in the proteasome [12]. Thus nuclear export minimizes the ability of FoxO3a to promote transcription of its target genes, including p27^kip1^, p21^Cip1^ and the retinoblastoma protein family member p130 [9,13]. In summary, direct effects of the downstream PDGFRα-signaling target Akt, and indirect effects of progressive FoxO3a phosphorylation by Akt combine to limit growth suppression by p27^kip1^.

## Methods

### Animals

Mice bearing the PDGF-Rα-GFP construct have been described [7,14,15]. Production and nuclear localization of green fluorescent protein (GFP) is under the control of the endogenous *pdgf-r*α promoter. GFP expression in the PDGF-Rα-GFP mice spatially and temporally recapitulated endogenous *pdgf-r*α expression, and the intensity of GFP-fluorescence correlated with the abundance of immunoreactive PDGFRα [14]. The mice used in this study carried one *pdgf-r*α*-*GFP allele (which does not encode for active PDGF-Rα) and one functional *pdgf-r*α allele. The heterozygous mice are phenotypically identical to wildtype (GFP^-^) mice, except for nuclear GFP, which enables their identification [14]. Mice expressing Cre-recombinase, which was targeted to the endogenous transgelin gene, B6.129S6-Tagln^tm2(cre)Yec^/J, stock number 006878 (Jackson Laboratories, Bar Harbor, ME) were bred with mice with LoxP-flanking exons 1 and 4 of the *pdgfR*α gene B6.Cg-Pdgfra^tm8Sor^/EiJ Jackson Laboratories stock number 006492 to obtain mice which contained at least one transgelin allele (TGCre +/−) and two LoxP-flanked *pdgfR*α alleles (PDGFRαF/F). Mice were genotyped using DNA isolated from tail biopsies and PCR, as recommended by Jackson Laboratories and were euthanized at postnatal day 8 or 12. Mice containing one transgelin (TG) Cre allele are denoted as TGCre+/−, one or two LoxP-flanked PDGFRα-allele(s) are denoted as PDGFRαF/- or PDGFRαF/F, respectively. Mice bearing the Cre-mediated deletion of PDGFRα are designated as TGCre+/−;PDGFRαF/F. The right lungs were uniformly inflated using 50 μl of fixative containing agarose per gram body weight, and fixed for 6 hours for laser scanning confocal microscopy (LSCM) [3]. The left lungs were inflated with fixative and then fixed overnight in 4% paraformaldehyde at 4^o^ and embedded in paraffin. Mice were housed in Thoren cages with access to food and water *ad libitum* in a thermally regulated environment and a 12-hour light/dark cycle. Protocols for animal use were approved by the Iowa City Veterans Affairs Medical Center animal use committee [1]. Lung fibroblasts were isolated from heterozygous mice on postnatal day 12 or 14 day old mice using a previously reported method [1].

### Antibodies

*Immunofluorescence and laser scanning confocal microscopy (LSCM)* p27^Kip^: Cell Signaling #3686, 75D8, rabbit monoclonal antibody (Mab) IgG 1:800 dilution. αSMA: Sigma-Aldrich (St. Louis MO), clone 1A4, mouse IgG2a, 1:400 dilution. FoxO3a: Millipore #07-1719, 1 μg/ml. Ki67: Dako rat anti-mouse Ki67 Mab clone TEC-3, 1:200 dilution. *Immunofluorescence of cultured primary mouse LF* FoxO3a: Cell signaling # 2497, 75D8 rabbit MAb. *Western immunoblotting* FoxO3a: Millipore #07-1719 1 μg/ml. anti- p(S253) FoxO3a Millipore rabbit polyclonal IgG, 06–953 1 μg/ml, directed at the N-terminus of FoxO3a. Secondary antibody was horse radish peroxidase-labeled goat anti rabbit IgG diluted 1:2000. Mouse anti-cleaved Asp214 poly-ADP ribose polymerase (PARP-1, 0.5 μg/ml, BD Biosciences, San Jose, CA).

### Analysis of DNA in mouse LF using flow cytometry

Our procedure for isolation LF has been published [7]. Using flow cytometry we have not found cytokeratin 18+ alveolar epithelial cells in the GFP+subpopulation and by this criterion, epithelial cells comprised only 2.7 ± 3.0 or 8.1 ± 6.0% (mean±SD) of the GFP- population at P4 and P12. Fewer than 6% of the GFP + cells stained for any of the markers for either for epithelial, endothelial, or macrophages. For analyzing the cell cycle distribution, freshly isolated mouse LF were fixed for 20 minutes at 4° with 0.5% paraformaldehyde, washed and then fixed for 1 h at 4° with 70% methanol that had been cooled to -20°. After washing, the LF were resuspended in 0.145 M NaCl, 0.0015 M KH2PO4, 0.0027 M KCl, 0.0086 M Na2HPO4, pH 7.4 (PBS) and incubated for 30 minutes with 20 Kunitz units of RNase A at 25°, and then with 35 μg/ml propidium iodide for 1 h at 4°. The cells were subjected to flow cytometry using a LSR II instrument (BD Biosciences, San Jose, CA) [7]. A minimum of 20,000 gated events were analyzed. The background fluorescence from the IgG isotype controls was subtracted prior to calculating the proportions of the different fibroblast populations. The data were analyzed using CellQuest Software (BD Biosciences) [7]. The proportions of LF in either the GFP + or the GFP- residing in G_0_/G_1_, S, or G_2_/M were calculated based on the intensity of PI-fluorescence using ModFitLT (Verity Software, Topsham ME). The phases of the cell cycle were distinguished from a plot of forward scatter vs. PI area, after correcting for PI-width to exclude aggregates of LF.

### Analysis of *foxO3a* gene expression using real-time quantitative PCR (RT-qPCR)

Total RNA was isolated using Tri-Reagent (Sigma-Aldrich, St. Louis, MO), subjected to reverse transcription, and *foxO3a* (Mm01185722.m1), and *β-2-microglobulin* (Mm00437762.m1) mRNA were quantified using Taqman Gene Expression Assays [7]. Four independent experiments were performed. Values for *foxO3a* expression were normalized to β-2-microglobulin using the 2^(−ΔΔCT)^ relative quantification method [16] and expressed relative to the 2^(−ΔΔCT)^ value at P4. The β-2-microglobulin Taq-man probe was used for normalization because we have not observed variation in β2 M mRNA, which appeared to be related experimental treatment conditions or developmental age. The mean±SEM Ct values for β2 M for the mice at P4, P8, and P12 were 19.66 ± 0.23, 19.18 ± 0.30, and 18.94 ± 0.38, respectively.

### Phospho FoxO3a Western Immunoblotting

After washing with 25 mM HEPES, pH 7.4, 150 mM NaCl, 2 mM Na_3_VO_4_ at 4°, the cell layers were lysed with 10 mM Tris–HCl, pH 7.4, 5 mM EDTA, 50 mM NaCl, 50 mM sodium fluoride, 1% Triton X-100, 1 mM phenylmethylsulfonyl fluoride, 2 mM Na_3_VO_4_, and 20 μg/ml aprotinin [1]. Equal quantities of protein were subjected to SDS-PAGE using a 6% acrylamide gel, and the proteins were transferred to nitrocellulose. Anti-phospho (S253) FoxO3a and anti-FoxO3a were diluted 1:1000. After washing, the primary antibodies were detected using goat anti-rabbit peroxidase and enhanced chemiluminescence (ECL, GE Healthcare, Piscataway, NJ). Fluorography was performed and the film was imaged and analyzed using a Quantity-One Imaging system (BioRad, Hercules, CA). The densities of phospho-FoxO3a were normalized to the density of FoxO3a for each lane, to account for differences in the amounts of protein which were loaded.

### Effects of PDGF-A on the distribution FoxO3a in cultured LF

LF, which had been isolated from PDGFRα-GFP+mice, were cultured on cleaned glass coverslips which had been coated with 8 μg/ml vitronectin to promote cell adhesion. The mouse LF were allowed to adhere to the coverslips for 1 hour, non-adherent cells were removed by washing and the adherent LF were cultured overnight in Ham’s F-12 medium containing 10% FBS. The next morning the LF were washed and the medium was changed to Opti-MEM for 12 hours prior to adding 50 ng/ml PDGF-A to some of the coverslips. The LF were cultured for an additional 12 h, washed with PBS and fixed for 20 min at 4° with 2% paraformaldehyde. After washing with PBS and water the coverslips were air dried and stored at 4° until they were immunostained. After permeabilization with 0.3% Triton X-100 and blocking with 2% normal goat serum, the coverslips were incubated overnight with rabbit anti-FoxO3a (Cell Signaling #2497, 1:200 dilution), anti-Ki67 (1:200 dilution), washed and then incubated with goat anti-rabbit IgG AlexaFluor 350 and goat anti-rat AlexaFluor 568 respectively, both at 1:2000 dilutions. The stained coverslips were mounted and imaged using an Olympus IX-81 microscope using the appropriate filter sets for A350, A568, and GFP. Nuclei and the cytoplasmic boundaries were identified using phase contrast optics and images using phase contrast and the three filters were captured with an Olympus XM-10 camera at a 1024 X 1024 pixel density. The images were merged using Cell-Sens® Software (Olympus, Center Valley, PA) and nuclei containing Ki67 or FoxO3a were enumerated in the GFP+and GFP- sub-populations.

### Laser scanning confocal microscopy (LSCM): tissue preparation and imaging

Lungs from mice at P4, P8 or P12 were uniformly inflated (50 μl per g body weight), fixed, and sectioned at 7 μm intervals for p27^kip1^ and FoxO3a [3]. Quantification of nuclei containing p27^kip1^ or FoxO3a was done using lung tissue sections, which were 7 μm in thickness, because background staining was excessive in 50 μm sections. This limitation did not permit enumeration of nuclei within a known volume of tissue. Therefore we determined the proportions of p27^kip1^ nuclei within PDGFRα-expressing (GFP+) and PDGFRα-non expressing (GFP-) cells. Nuclei were stained with 1:15,000 dilution of PoPo3 (stock in DMSO, InVitrogen, Molecular Probes). Image of the stained sections were acquired from randomly selected fields using Zeiss LSM710 laser scanning confocal microscope at a 1024 X 1024 pixel density. Incubations with the primary antibodies were overnight at 4°. All secondary antibodies were AlexaFluor-labelled and used at a 1:2000 dilution. The laser intensities and detector gains were optimized at the beginning and remained constant for each channel throughout the imaging session. The following excitation/emission filters were used: a 488/492-536 nm band pass filter to detect GFP or AlexaFluor 488, a 547–590 nm band pass filter to detect PoPo3 or FoxO3a, and a 637–757 nm band pass filter for p27^kip1^.

### Cell culture

Primary cultures of mouse LF were established as described, except that after the 1 hour adherence step, the plates were washed three times with PBS and the primary cells were grown to ~90% confluence in Ham’s F-12 medium containing 10% FBS and 10 mM HEPES [7]. After washing the near confluent monolayers with PBS, the medium was changed to OptiMEM-1 supplemented with 2.5% FBS, penicillin, streptomycin, and with CaCl_2_ to a final calcium concentration of 3 mM for 16 h prior to adding PDGF-AA (concentrations shown in the figures).

### Analysis of images acquired using LSCM

*General considerations*: Because of elevated non-specific staining with the anti-FoxO3a and anti-p27 antibodies using 50 or 100 μm sections, 7 micron sections were used to enumerate nuclei containing one of these antigens. For the analysis of p27^kip1^, nuclei were counterstained with PoPo3 and for the analysis of FoxO3a, nuclei were stained with 4’,6-diamidino-2-phenylindole, diacetate (DAPI). Because the LSM710 did not have a UV laser, DAPI fluorescence was excited by a metal halide lamp (Exfo X-cite120XL Exfo Mississauga, ON, Canada) metal halide lamp, and emitted light was collected without pin-hole optics. The Zeiss czi-images were converted to 8-bit TIF images and the colors were merged into RGB TIF files using image J (National Institutes of Health). IP-Lab for Windows (BD-Scanalytics, San Jose, CA) was used to identify nuclei which contained p27^kip1^ or FoxO3a, using uniform segmentation criteria.

*Evaluation of nuclear p27*^*kip*^*in PDGFRα-GFP+mice*: For the analysis of p27, cells in the alveolar walls were classified as follows: nuclear GFP+(*pdgf-r*α expressing, green), PoPo3+ (all nuclei, red), p27^kip1^(AlexaFluor 647, pseudocolor blue) in nuclei without GFP (PoPo3, but without GFP) and p27^kip1^ in nuclei containing GFP (and stained with PoPo3). Five fields were analyzed per mouse using 3 mice at each age. Mice for each of the ages were included, and the LSM710 acquisition parameters were held constant for all ages within a staining and imaging cohort. Using uniform segmentation criteria and IP-Lab, the pixels-regions that contained (a) both red and blue, but not green, (nuclei which were p27^kip1^ + and GFP-), (b) red, blue, and green (nuclei which were p27^kip1^+ and GFP+), and (c) all nuclei (red). The nuclei of cells which were p27^kip1^ + GFP-, or p27^kip1^ +, GFP+nuclei were enumerated and expressed relative to the total of PDGFRα-expressing alveolar cells (GFP+) or other, non-expressing alveolar cells (GFP-). The percentages of nuclei in p27^kip1^+, GFP+or p27^kip1^+, GFP- groups were calculated for each field and a mean and SEM were determined for each mouse. The data from four mice were combined to obtain a mean and SEM for each age.

*Evaluation of nuclear FoxO3a in PDGFRα-GFP+mice*: Four mice and a total of 21 fields were analyzed at each age: P4, P8 and P12 and one mouse from every age was included in each of the four staining sessions. Rabbit anti-FoxO3a was identified using goat-anti rabbit IgG AlexaFluor 647 (blue pseudocolor), nuclei were counterstained with DAPI (and assigned either a green or blue pseudocolor), and Ki67 was identified using goat anti-rat AlexaFluor 568 (red). Images, which had been captured using the LSM710, were exported in an 8-bit TIF format and converted to RGB images using ImageJ. The RGB images were analyzed using IP-Lab using uniform segmentation criteria. To identify Ki67 or FoxO3a in GFP- nuclei DAPI was pseudocolored green and GFP was not included in the color map. The pixel areas containing green and red (Ki67) or green and blue (FoxO3a) above the specified thresholds were considered Ki67+, GFP- nuclei. To analyze Ki67 and FoxO3a within GFP+nuclei DAPI was excluded and the pixel areas containing GFP (green), FoxO3a (blue) and Ki67 (red) were ascertained. To determine all pixels overlying nuclei, GFP, Ki67 and FoxO3a were excluded and the pixel area containing DAPI was ascertained. Likewise to ascertain the GFP+nuclear area, after excluding FoxO3a and Ki67, the pixel area containing both GFP (green) and DAPI (blue pseudocolor) were ascertained. The pixel area of alveolar nuclei containing Ki67 or FoxO3a but not GFP were expressed relative to the pixel area of DAPI-stained nuclei lacking GFP. Likewise, the pixel area of alveolar nuclei containing Ki67 or FoxO3a, as well as GFP, was expressed relative to the pixel area of DAPI-stained nuclei also containing GFP.

*Evaluation of nuclear p27*^*kip*^*in TGCre; PDGFRαFlox mice*: Because these mice do not contain the GFP insertion into *pdgfR*α, we used αSMA-FITC to identify regions where PDGFRα-expressing would be expected to reside. p27^kip1^ was stained as described for lungs from *pdgfR*α -GFP mice and PoPo3 was used to stain all nuclei. The mice were euthanized at P12 and 5 fields were analyzed per mouse using 4 mice TGCre+/−;PDGFRαF/F or littermate controls (bearing only one LoxP-flanked allele and or no Cre-alleles). Mice from both control and LoxP-flanked groups were included in every staining cohort, and the LSM710 acquisition parameters were held constant within a staining and imaging cohort. Uniform segmentation criteria were used to uniformly identify collections of pixels that contained both p27^kip1^ and PoPo3 (nuclei containing p27^kip1^). The cytoplasm around all nuclei was examined to determine whether it contained filamentous structures that stained with anti-αSMA. Nuclei which contained p27^kip1^ and were adjacent to were classified as p27^kip1^+, αSMA+, and those which were not adjacent to αSMA were classified as p27^kip1^+ and αSMA -. The enumerated nuclei of p27^kip1^+, αSMA+or p27^kip1^+, αSMA– cells were expressed relative to the total nuclei which were (αSMA +) or were not adjacent to αSMA, respectively.

*Analysis of cleaved poly-ADP ribose polymerase (PARP) in TGCre; PDGFRαFlox mice*: Lungs from TGCre+/−;PDGFRαF/F and littermate control mice ages P8 or P12 were uniformly inflated, fixed, and 100 μm cryosections were used for immunostaining and confocal microscopy. The sections were permeabilized with 0.3% Triton X-100 and 0.5 mg/ml 2.4 g2 rat-anti mouse Fc-receptor was added to block mouse Fc receptors. The primary antibody was mouse anti-cleaved PARP, the secondary antibody was goat anti-mouse IgG-AlexaFluor 647, and nuclei were counterstained with PoPo3. Anti-αSMA-FITC was used to locate regions where mesenchymal cells would be expected to reside. Three Z-stacks for each of 3 different portions of the tissue were imaged 1.5 at μm intervals for each mouse and sections were analyzed at 3 different z-levels. The starting z-level was in the center of the tissue and images were acquired in the 9 μm above and below this level. Comparisons were made at the same z-level for all lungs to normalize for differences in laser-penetration at different tissue depths. Uniform segmentation criteria were applied to identify nuclei which contained PARP-staining nuclei (more than 75% of the pixels in the nuclei met the blue segmentation criterion). Data were expressed as the ratio of cleaved PARP-containing nuclei to the total nuclei in the field. Three mice were used at each age for each genotype and the mean±SEM proportions of nuclei were calculated.

*Analysis using the proliferation marker Ki67* Lungs from TGCre+/−;PDGFRαF/F and littermate control mice at P8 were inflated, fixed and 100 μm sections were prepared. The tissues were incubated with rat anti-mouse Ki67 (DAKO, clone TEC-3, 18.5 μg/ml) for 1.5 h at 25°, followed by washing, and then with goat anti-rat IgG-A647 and anti-αSMA-FITC. Nuclei were counterstained with PoPo3. Z-stacks were acquired at 2.5 μm intervals over 42 μm and nuclei were enumerated using the optical fractionator. Nuclei were classified as staining for Ki67 or only PoPo3 and as adjacent or not adjacent to αSMA. The data from four mice of the same genotype were pooled.

### Evaluation of alveolar surface area in TGCre+;PDGFRαF/F and littermate controls

At P12, lungs were uniformly inflated by intratracheal instillation of 50 μl of 15% picric acid, 1% paraformaldehyde, 1% low melting point agarose in 0.1 M sodium-phosphate pH 7.2 per g body weight. After the agarose hardened, the lungs were removed from the thorax, their volume was measured by displacement, and the left lung was immersed in 4% paraformaldehyde for 20 hours. The right lungs were fixed for 6 h in 15% picric acid, 1% paraformaldehyde in 0.1 M sodium-phosphate pH 7.2, and the agarose was removed by several washes in PBS at 50° and used for confocal microscopy. The left lungs were embedded in paraffin, 4.5 mm sections were stained with hematoxylin and eosin. The surface area of the alveoli and alveolar ducts was determined (cycloids for Sv stereological probe, units μm^-1^) and multiplied by the displacement volume [1].

### Statistical methods

Data were expressed as the mean±SEM of the number of different mice that were used or the number of different experiments that were performed using cell cultures [17]. Analysis of variance (either 1 or 2-way as indicated in the figure legends) was performed using Systat (Chicago, IL) and Student’s t-test using Microsoft Excel. Additional details describing how the data from different images or image stacks were combined appears in the method for a particular stereological procedure. Post-hoc tests are described in the figure legends. Values of p < 0.05 were considered significant.

## Results

### A lower fraction of PDGFRα-expressing than non-expressing LF enter S-phase at P8 and P12

We previously showed that at P12 a smaller proportion PDGFRα-GFP+LF, compared to their GFP- counterparts, contained Ki67 a marker of proliferation [7]. By analyzing the distribution of DNA staining with propidium iodide, we have now observed that fewer freshly isolated GFP+than GFP- LF make the G_1_ to S transition, suggesting that the mechanism(s) responsible for this difference involve(s) proteins, which regulate this transition (Table 1). Because the expression of GFP is driven by PDGFRα, the mechanism also involves PDGF-mediated signaling through PDGFRα.

**Table 1:** **PDGFR**α**-expressing differ from non-expressing lung fibroblasts (LF) in cell cycle S-phase entry**

**Cycle stage**	**Age**		**P8**			**Cycle stage**	**Age**		**P12**		
	**GFP-**			**GFP+**			**GFP-**			**GFP+**	
	mean	SEM		mean	SEM		mean	SEM		mean	SEM
**G**_**0**_**/G**_**1**_	90.93 ±	0.64		95.40 ±	0.32	**G**_**0**_**/G**_**1**_	91.99 ±	0.69		97.97 ±	0.04
**S**	6.39 ±	0.48		2.65 ±	0.23*	**S**	5.68 ±	0.34		0.71 ±	0.10*†
**G**_**2**_**/M**	2.30±	0.17		1.95 ±	0.20	**G**_**2**_**/M**	2.13 ±	0.36		1.35 ±	0.13

Flow cytometry was used to separate freshly isolated *LF*, obtained at postnatal day 8 (P8) or 12 (P12) into sub-populations based on *PDGFR*α-gene expression and the presence of p27^kip1^. The proportions of *PDGFR*α-expressing (*GFP+*) in the S and G_2_/M phases were lower than in their non-expressing (*GFP-*) counterparts at both P8 and P12 (*, P < 0.05 2-way ANOVA). The proportions of LF in S-phase within the *GFP +* sub-population differed significantly between P8 and P12 (*) P < 0.05, compared to *GFP-; (†) P < 0.05 P12 GFP +* compared to P8. 2-way *ANOVA*, Student-Newman Keuls post-hoc test.

### p27^kip1^ is preferentially localized to the nucleus of PDGFRα-expressing alveolar cells at P12

**Figure 1:**
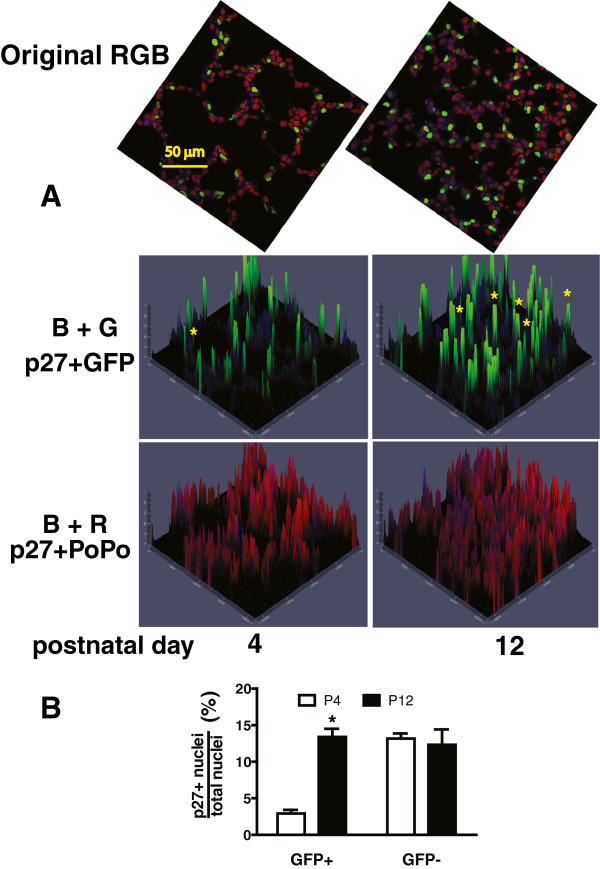
**A larger proportion of pixels in the nuclei of PDGFRα-GFP expressing alveolar cells contain p27**^**kip1 **^**at P12.** Lung tissues from PDGFRα-expressing (GFP+) mice were uniformly inflated and analyzed for nuclear p27^kip1^. **(A)** Representative microscopic fields are shown at postnatal days 4 and 12 (scale bar 50 μm). The top images show merged blue (p27^kip1^, AlexaFluor 647, green (GFP in nuclei of PDGFRα-expressing LF), and red (PoPo3 nuclear stain). Contour plots show the co-localization of p27^kip1^ in GFP+nuclei (BG) and p27^kip1^ in the nuclei of other alveolar cells, and which lack GFP (BR). Yellow asterisks mark PDGFRα-GFP+nuclei that contain p27^kip1^, which are more numerous at P12. **(B)** Analysis of 5 images, similar to those shown in **(A)** obtained from each of 4 separate mice (20 images) at postnatal days 4 (open) and 12 (closed) bars. The percentage of p27^kip1^-containing pixels in non-expressing (GFP-) alveolar cells did not vary with age, whereas the proportion was higher in the PDGFRα-expressing (GFP+) alveolar cells at P12. (*, p < 0.01, 2-way ANOVA analyzing GFP+population), Student-Newman Keuls post-hoc test.

Regulation of S-phase entry is dependent on whether p27^kip1^ resides in the nucleus or cytoplasm. Translocation of p27^kip1^ from the nucleus (where it inhibits CDKs) to the cytoplasm (where it is degraded) is also altered by PDGF-mediated signaling [18]. Therefore we examined the intracellular location of p27^kip1^ in the alveolar region of lungs from PDGFRα-GFP+mice. We observed that the proportion of PDGFR-expressing LF (marked by their nuclear GFP) which contain nuclear p27^kip1^ is significantly higher at P12, whereas this proportion does not change in the remaining alveolar cells, which lack the GFP-marker of PDGFRα-gene expression and whose nuclei only exhibit staining with PoPo3 (Figure 1). These data indicate that PDGFRα-mediated signaling may regulate the intracellular location of p27^kip1^ and that regulation changes during septal formation. We studied this more directly using cultured LF to examine how PDGF-A alters the intracellular location of FoxO3a, an important regulator of p27^kip1^ translocation.

### PDGF-A increased FoxO3a phorphorylation and translocation to the cytoplasm

**Figure 2:**
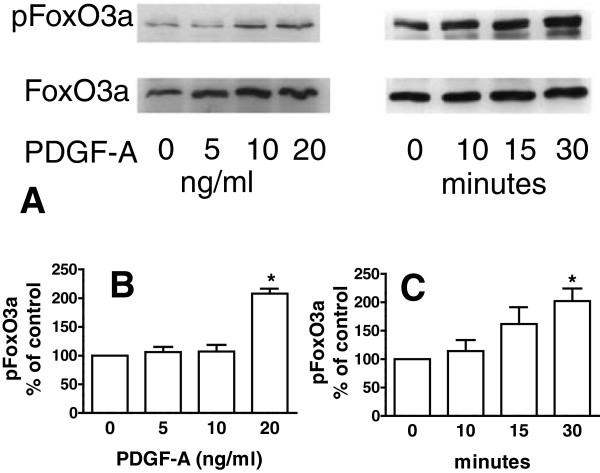
**Phosphorylated FoxO3a (pFoxO3a) in cultured lung fibroblasts (LF) was increased by PDGF-A.** After reducing the concentration of fetal bovine serum to 2% for 12 h, primary cultures of LF (which were obtained from mice at P12) were exposed to PDGF-A for 20 minutes at the concentrations shown or to 20 ng of PDGF-A per milliliter for the indicated times. **(A)** Representative immunoblots showing pFoxO3a, which were re-probed to assess total FoxO3a. Scanned images shown in **(A)** and 2 additional immunoblots, for both the dose–response **(B)** and time course **(C)**, using different primary cell isolations are shown as mean±SEM. (*) p < 0.05, 2-way ANOVA, Student-Newman Keuls post-hoc test.

The nuclear abundance of p27^kip1^ is determined both by its accumulation, mediated in part by transcriptional effects of FoxO3a, and by its egress from the nucleus. Phosphorylation of p27^kip1^ by Akt promotes its translocation from the nucleus to the cytoplasm [19,20]. We hypothesized that PDGFRα-signaling decreases nuclear p27^kip1^ through phospho-Akt and phospho-FoxO3a. Because Akt-phosphorylation is initiated from multiple pathways, it is not feasible to use phospho-Akt as a unique marker of PDGFRα-signaling. Therefore, we focused on FoxO3a-phosphorylation, as a target of PDGFRα-signaling, because it directly alters the fate of p27^kip1^. Using cultured LF and Western immunoblotting, we found that PDGF-A increased the phosphorylation of serine 253 in FoxO3a in a time and concentration related manner (Figure 2).

**Figure 3:**
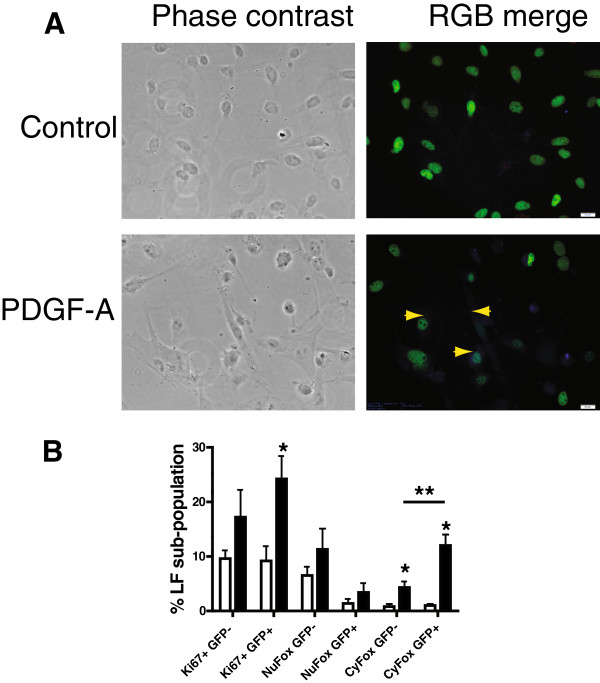
**PDGF-A promotes localization of FoxO3a to the cytoplasm.** LF isolated from PDGFRα-GFP+mice at P12 and cultured, were exposed to 50 ng PDGF-A per ml for 12 h prior to fixation. Immunostaining was performed and FoxO3a was visualized with AlexaFluor 350 (blue), Ki67 was visualized with AlexaFluor 568 (red) and GFP (green). The cellular and nuclear perimeters were visualized using phase-contrast optics (scale bar 20 μm). **(A)** Representative fields show more cytoplasmic FoxO3a (yellow arrows) in PDGF-A exposed than in unexposed control cultures. **(B)** Combined data from 4 experiments using different cell-isolations comparing nuclear (Nu) and cytoplasmic (Cy) staining of FoxO3a (Fox) and Ki67 in PDGFRα-expressing (GFP+) and non-expressing (GFP-), LF which were exposed to PDGF-A (solid bars) or remained unexposed (control, open bars). Mean±SEM, (*) p < 0.05 PDGF-A compared to control. (**) p < 0.05 comparing cytoplasmic FoxO3a in GFP- and GFP+LF, 1-way ANOVA, Student-Newman Keuls post-hoc test.

Nuclear FoxO3a directly stimulates p27^kip1^-gene expression, increasing nuclear p27^kip1^, resulting in CDK-inhibition [21]. PDGF-A-signaling through PDGFRα stimulates the phosphorylation of FoxO3a, leading to its translocation from the nucleus to the cytoplasm, and suppresses the stimulation of p27^kip1^ gene expression. Therefore the translocation of FoxO3a can serve as a surrogate marker for the effects of PDGF-A and PDGFRα-mediated effects. Using cultured LF we found that PDGF-A caused a significantly greater increase in the proportion of LF with cytoplasmic FoxO3a in PDGFRα-GFP+compared to GFP- LF, consistent with a PDGFRα-mediated effect (Figure 3). This was accompanied by a significant increase in the fraction of Ki67+ cells in the GFP+LF sub-population after treatment with PDGF-A. A smaller but significant increase in cytoplasmic FoxO3a was also observed in GFP- LF after exposure to PDGF-A. This increase may reflect the reduced sensitivity (compared to LSCM or flow cytometry) of the epifluorescence microscope to low level GFP-emission, so that weakly GFP+LF were scored as GFP-.

### FoxO3a gene expression in neonatal mouse LF varies with age

**Figure 4:**
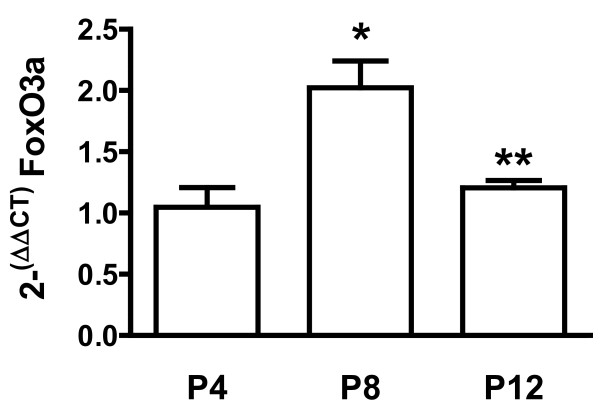
**FoxO3a gene-expression is increased at P8.** RNA from freshly isolated lung fibroblasts at postnatal days 4 (P4), 8 (P8) and 12 (P12) was quantified using real-time PCR and Taq-Man probes, normalizing to P4. Mean±SEM, n = 4 at P4 and n = 3 at both P8 and P12. (*) P < 0.05 compared to P4. (**) p < 0.05 compared to P8.

Using quantitative real-time PCR, we found that FoxO3a gene expression was significantly higher at P8 than at P4 and that there was a significant decrease by P12 (Figure 4). The discordance between FoxO3a-gene expression and nuclear p27 suggests that that *foxO3a* gene-transcription does not determine its effects on the abundance of nuclear p27^kip1^, which may be more directly related to the intracellular location of previously synthesized FoxO3a.

### A larger proportion of PDGFRα expressing LF contain nuclear FoxO3a at P12

**Figure 5:**
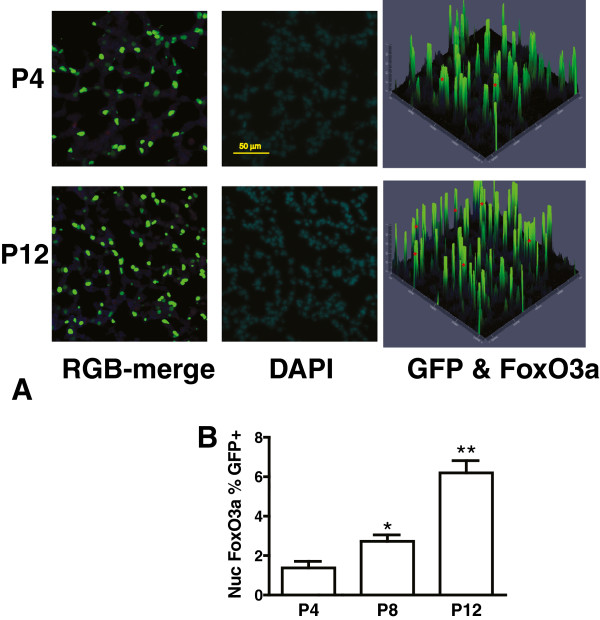
**Nuclear FoxO3a in PDGFRα-expressing lung fibroblasts (LF) increases with age. (A)** Uniformly inflated lung tissues from PDGFRα-GFP expressing mice (GFP+, green nuclei) at postnatal days (P) P4, P8 and P12 were stained for Ki67 (AlexaFluor 568, red), FoxO3a (AlexaFluor 647, blue), and with DAPI (all nuclei). Representative images are shown in left panels for P4 and P12 (P8 not shown) and the right panels are contour plots showing pixels (red asterisks) which contain FoxO3a (blue) in GFP+(green) nuclei. Middle panels show all nuclei, stained with DAPI (scale bar 50 μm). **(B)** Histograms showing that the mean±SEM percentages of nuclei in PDGFRα-expressing (GFP+) LF which contain FoxO3a increases with postnatal age. (*) p < 0.05 comparing P8 to P4, (**) p < 0.01 comparing P12 to P8 and P4, 2-way ANOVA, Student-Newman Keuls post-hoc test. Lungs from 3 separate mice were used at each age, analyzing 6 fields per lung.

If the intracellular location of FoxO3a is the major determinant nuclear p27^kip1^ at P12, then more FoxO3a should be found in the nucleus at P12 than at P8. Furthermore, if PDGFRα-signaling regulates FoxO3a, then the distribution of FoxO3a may differ between PDGFRα-expressing (GFP+) and non-expressing (GFP-) alveolar cells. The proportion of GFP+alveolar cells containing nuclear FoxO3a was maximal at P12, and the lowest at P4, when we have previously shown that a larger fraction of these cells contain the Ki67 antigen (Figure 5). These data show that at P12, when fewer PDGFRα-expressing LF enter S-phase, there is maximal nuclear co-localization of FoxO3a and p27^kip1^, consistent with growth arrest at the G_1_/S boundary. This could prevent further expansion of the PDGFRα-expressing LF population. We have previously shown that PDGF-A signaling protects PDGFRα-expressing LF from apoptosis, which without further expansion would cause a decline in the PDGFRα-expressing LF sub-population [1].

### Conditional deletion of PDGFRα decreases proliferation and increases apoptosis in the nuclei of alveolar cells adjacent to αSMA

**Figure 6:**
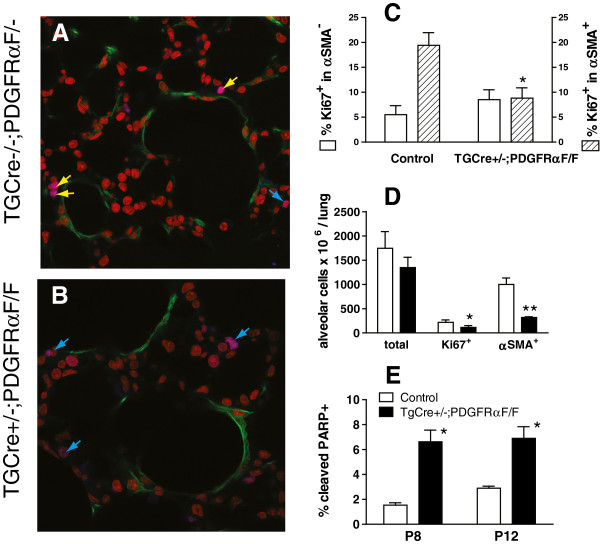
**Deletion of PDGFRα reduces proliferation and increases apoptosis of alveolar cells proximate to αSMA.** Lungs from control and PDGFRα-gene deleted mice were examined for Ki67 antigen (marker of proliferation) at P8 and for cleaved PARP (marker of apoptosis) at P8 and P12. Representative images showing nuclear Ki67 in TGCre−/−;PDGFRαF/- (control, **A**) and a TGCre+/−;PDGFRαF/F (gene deleted, **B**) mice. Alveolar cells were classified as adjacent to (yellow arrows) or separated from (blue arrows) bundles of FITC-αSMA (green) and Ki67-containing (blue) cells were expressed as percentage of alveolar cells which were adjacent to or separated from αSMA. **(C)** Combined data from 4 mice of each genotype showing Ki67+ proximate to (striped bar) or separate from αSMA (open bar). Mean±SEM, n = 4, (*) p < 0.05 comparing Ki67 proximate to αSMA for the two genotypes. **(D)** Alveolar cells were enumerated and classified as Ki67-containing (Ki67+) or αSMA-associated in control (open) or TGCre+/−;PDGFRαF/F (solid bar) mice at P8 (mean±SEM, n = 4). (*) p < 0.05, (**) p < 0.01 comparing control and gene-deleted mice at P8, 2-way ANOVA on ranks, Student-Newman Keuls post-hoc test. **(E)** Alveolar cells containing cleaved-PARP in lung tissues from control (open) or gene-deleted mice (solid) bars mice were enumerated at P8 and P12 and expressed as a percentage of all alveolar cells. (mean±SEM, n = 3 mice for each genotype at each age). (*) p < 0.01 comparing control and gene-deleted mice, 2-way ANOVA on ranks, Student-Newman Keuls post-hoc test.

PDGFRα-expressing mesenchymal cells are located near αSMA and elastic fibers, at points where there is increased mechanical tension [3]. We examined whether PDGFRα-gene deletion depleted alveolar cells at these locations by diminishing proliferation and/or increasing apoptosis. Transgelin (a marker of a smooth muscle-like phenotype) was used to direct Cre-mediated deletion of PDGFRα in alveolar myofibroblast. We stained tissues for αSMA to identify areas where myofibroblasts should reside. Figure 6 shows that PDGFRα-gene deletion specifically reduced the proportion of nuclei which were Ki67+ (A-D) and increased the proportion of nuclei containing cleaved poly ADP-ribose polymerase (PARP, a marker of apoptosis) in cells adjacent to αSMA (E). These data corroborate our finding that disruption of PDGFRα-kinase activity depletes the PDGFRα-expressing LF sub-population and establishes that depletion occurs in regions where αSMA would normally be most abundant [3].

### Alveoli from mice lacking PDGFRα contain more nuclear p27^kip1^

**Figure 7:**
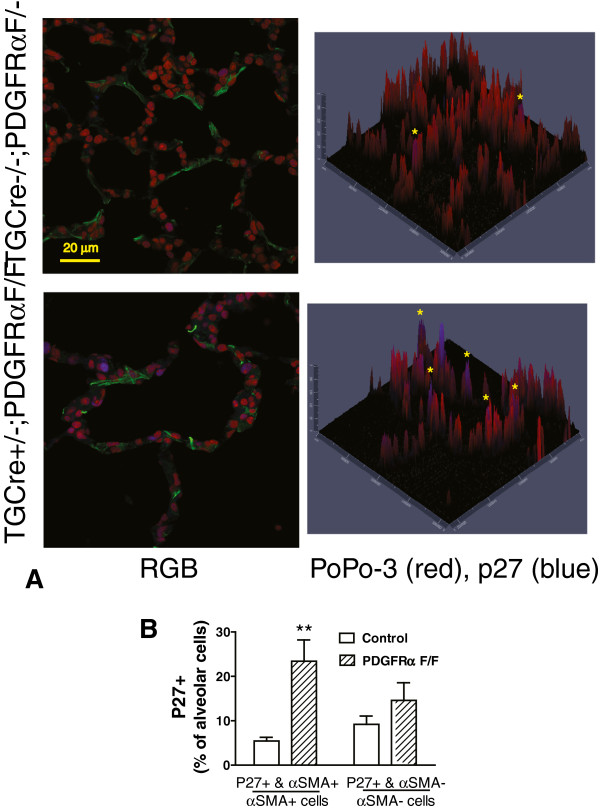
**Targeted deletion of PDGFRα increases the proportion of nuclei in alveolar cells which contain p27**^**kip1**^**. (A)** Lungs from TGCre+/−;PDGFRαF/F mice, or littermates which did not contain 2 LoxP-flanked PDGFRα-alleles, were uniformly inflated and fixed at postnatal day 12 (P12). Staining with FITC-conjugated anti-ACTA2 (αSMA, green), p27^kip1^ (p27, blue), and all nuclei (PoPo3, red) is shown (scale bar 20 μm). Contour plots show co-localization of p27^kip1^ (blue) in nuclei (PoPo3, red). **(B)** Histograms comparing the mean±SEM fraction of alveolar cells either containing (αSMA+) or not containing vimentin (αSMA-), which also contain nuclear p27^kip1^ in lungs from 4 PDGFRα-deleted (hatched bars) and 4 control mice (open bars) at P12. (**) p < 0.01, comparing nuclear p27^kip1^ in αSMA-containing LF in control and gene-deleted mice, 2-way ANOVA on ranks, Student-Newman Keuls post-hoc test.

**Figure 8:**
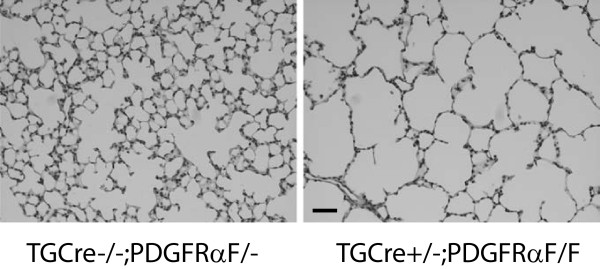
**Targeted deletion of PDGFRα decreases alveolar surface area.** With a single endogenous transgelin (TG) allele (+/−) driving Cre-recombination in lung fibroblasts (LF), PDGFRα flanked by 2 LoxP sites (Flox/Flox, F/F) gene expression was inactivated. This reduced the surface area of the alveolar region compared to the control, TGCre+/−;PDGFRα−/− (no floxed alleles, scale bar 50 μm).

We also examined the proportion of alveolar cells which contain nuclear p27^kip1^ in mice with the targeted deletion of PDGFRα in alveolar cells. We observed a significant increase in the quantity of alveolar cells containing nuclear p27^kip1^ adjacent to αSMA, compared to their littermates which retain PDGFRα (Figure 7). Deletion of PDGFRα in the sub-population of alveolar cells, which express transgelin, significantly decreased the internal surface area of the alveolar gas-exchange structures (Figure 8). At P12, the mean alveolar surface area for littermate controls, without the deletion was 75.5 ± 6.6 compared to 38.9 ± 4.9 cm^2^ for mice with the PDGFRα-deletion (mean SEM, n = 4 for each group, P < 0.01 Student’s t-test, unpaired variables). The Sv (units μm^-1^) was multiplied by the displacement volume for each mouse [22]. The mean volumes for control and TGCre+;PDGFRαF/F mice were 0.419 ± 0.018 and 0.441 ± 0.36 (mean±SEM), respectively.

## Discussion

As secondary septal elongation decelerates at P12, there is a preferential decrease (assessed by the Ki67antigen using FACS) in proliferation of the PDGFRα-expressing LF sub-population [1]. We have now shown that the transition from the G_1_/G_0_ to S-phase is diminished at P12 in PDGFRα-expressing LF (Table 1). At P12, nuclear p27^kip1^ accumulates along with its transcriptional activator FoxO3a in PDGFRα-expressing fibroblasts (Figure 1). In contrast PDGF-A mobilizes more FoxO3a from the nucleus to the cytoplasm in PDGFRα-expressing fibroblasts, which also contain more Ki67 than their non-expressing LF counterparts (Figure 3B). Taken together these findings suggest that FoxO3a, a known downstream target of PDGFRα signaling, is an important regulator of cell-cycle kinetics in PDGFRα-expressing LF. When PDGFRα is absent, there is a significant increase in nuclear p27^kip1^, which is accompanied by a decrease in proliferation and an increase in apoptosis in cells proximate to αSMA (Figure 6). These observations are consistent with diminished FoxO3a-phosphorylation and translocation to the cytoplasm, resulting in unopposed accumulation of nuclear p27^kip1^ (Figure 7). This leads to diminished secondary septal elongation resulting in a marked decrease in alveolar surface area (Figure 8).

*Alternative regulators of p27*^*kip1*^*and FoxO3a phosphorylation and S-phase entry:* Other factors may influence the abundance and location of p27^kip1^. Signaling by insulin-like growth factor-1 (IGF-1) or insulin also stimulates Akt-mediated phosphorylation of p27^kip1^ and FoxO3a, and increases LF proliferation [23]. In the lung, PDGF-A is probably not the exclusive regulator of FoxO3a phosphorylation and p27^kip1^-mediated S-phase entry in PDGFRα-expressing LF. Studies using mouse embryonic fibroblasts showed that fibroblast growth factor-2 can indirectly stimulate PDGFRα-phosphorylation through Src-family kinases, in the absence of PDGF-A [24]. However, S-phase entry and the nuclear abundance of p27^kip1^ and FoxO3a are regulated differently in PDGFRα-expressing compared to non-expressing LF, and in mice bearing the PDGFRα gene-deletion, indicating that PDGFRα-mediated signaling is an important determinant of cell-cycle progression in cells that express this receptor.

It is also likely that p27^kip1^ is not the exclusive regulator of S-phase entry in PDGFRα-expressing LF. Others have shown that PDGFRα-expressing LF predominantly lack Thy-1, and demonstrate characteristics of myofibroblasts. In the presence of 1% fetal bovine serum, Thy-1 negative human LF were more proliferative than their Thy1-positive counterparts [25]. Similarly, vimentin-containing lung cells obtained from mice bearing a Thy-1 gene-deletion were more proliferative than a comparable population obtained from mice without the deletion [26]. These data indicate that Thy-1 differentially regulates proliferation in PDGFRα-expressing and non-expressing LF. But whereas Thy-1 gene deletion increases proliferation, PDGFRα-gene deletion has anti-proliferative effects.

*The effects of p27*^*kip*1^*are more consequential in cells or conditions where PDGFR*α*-signaling normally occurs*: We found that nuclear p27^kip1^ is more prevalent in alveolar mesenchymal cells in mice which lack PDGFRα (TgCre+;PDGFRαF/F), compared to mesenchymal cells in mice with PDGFRα. This is accompanied by a decrease in proliferation and increase in apoptosis within the same population. Our prior studies showed a preferential decrease in the proliferation of *pdgfr*α-expressing, compared to non-expressing LF at P12, and that PDGFRα-kinase activity is required to maintain proliferation [1,7]. We have now found that p27^kip1^ preferentially accumulates in the nuclei of LF, which are sensitive to a reduction of PDGFRα-mediated signaling; and that this accumulation is augmented in the absence of PDGFRα-mediated signaling. Therefore PDGF-A is required not only for establishing and expanding a myofibroblast precursor, but also for maintenance of myofibroblasts during maximal secondary septation.

*The intracellular location of FoxO3a and p27kip1 is more consequential than their abundance*: We observed that FoxO3a mRNA was lower at P12 than at P8, whereas a larger proportion of PDGFRα-GFP+LF contained nuclear FoxO3a and p27^kip1^ at P12. There are several reasons for this observation. (a) RNA was isolated from both LF which contain and those which lack PDGFRα, potentially obscuring differences between the two LF subpopulations (b) The intracellular location of FoxO3a (determined post-translationally) more directly and potently alters nuclear p27^kip1^ than does the abundance of FoxO3a mRNA. And (c) at P8, when a larger proportion of PDGFRα-expressing LF are entering S-phase, the predominant location of FoxO3a may be cytoplasmic. We could not evaluate cytoplasmic FoxO3a in lung tissues using confocal microscopy, because we could not accurately demarcate cell-cell boundaries. The intracellular localization and abundance of p27^kip1^ are regulated by multiple pathways and in a complex fashion. Therefore although FoxO3a is particularly relevant to PDGFRα signaling, other factors likely regulate p27^kip1^. PDGF-A independent, Smad-dependent TGFβ-signaling may also increase nuclear p27^kip1^ by reducing its cytoplasmic ubiquitination and proteasomal degradation [27]. Likewise, the intracellular location of FoxO3a is dependent on not only phosphorylation but also on acetylation, which determines how readily adult mice develop emphysema after exposure to cigarette smoke [28,29].

## Conclusions

Secondary alveolar septation requires that PDGFRα-expressing LF, which are located where primary septa erupt at P4, expand in number, and relocate more distally as the secondary septa elongate. Boström and associates hypothesized that signaling through PDGFRα was required to establish the precursor population of LF in the primary septa which could then spread into the secondary septa [2]. Our studies demonstrate that sustained PDGFRα-signaling is required during secondary septal elongation to expand the number of PDGFRα-expressing LF. And when elongation nears completion, proliferation is curtailed as PDGF-A-initiated events (translocation of p27^kip1^ and FoxO3a into the cytoplasm) subside in LF expressing PDGFRα. This allows p27^kip1^ to accumulate in the nucleus, where its inhibition of cyclin-dependent kinases reduces progression from G_1_ to S-phase. Therefore, the expansion and regression of the PDGFRα-expressing, myofibroblastic, elastin producing, LF is tightly regulated. Therapeutic strategies for alveolar regeneration in humans need to carefully modulate both the onset and reduction in stimulation of the PDGFRα-expressing LF, to accommodate both septal elongation and thinning.

## Abbreviations

DAPI: 4’,6-Diamidino-2-phenylindole, diacetate; Akt: Akt, protein kinase B; p27kip1: CDK inhibitor 1B; CDK: Cyclin-dependent kinase; FoxO: Forkhead box factor O (other) transcription factors; Thy-1: Glycoprotein thymocyte differentiation antigen-1; GFP: Green fluorescent protein; LSCM: Laser scanning confocal microscopy; LF: Lung fibroblasts; PDGFRα-GFP: Mice bearing GFP-insertion regulated by endogenous PDGFRα-promoter; PARP: Poly-ADP ribose polymerase; PI3K: Phophoinositide 3-kinase; PDGF-A: Platelet derived growth factor; PDGFR: Platelet-derived growth factor receptor; P: Postnatal day; RGB: Red-green-blue; TG: Transgelin; αSMA, ACTA2: α-Smooth muscle actin.

## Competing interests

The neither author has any competing financial or non-financial interests.

## Authors’ contributions

SEM designed the experiments, performed the flow cytometry, analyzed and interpreted the data, and wrote the manuscript. DMM isolated the LF, isolated RNA and protein from cultured cells, performed the immunostaining and LSCM, and edited the manuscript. Both authors read approved the final manuscript.
